# Optimisation of treatment conditions for reducing *Shewanella putrefaciens* and *Salmonella* Typhimurium on grass carp treated by thermoultrasound-assisted plasma functionalized buffer

**DOI:** 10.1016/j.ultsonch.2021.105609

**Published:** 2021-05-26

**Authors:** Okon Johnson Esua, Jun-Hu Cheng, Da-Wen Sun

**Affiliations:** a School of Food Science and Engineering, South China University of Technology, Guangzhou 510641, China; b Academy of Contemporary Food Engineering, South China University of Technology, Guangzhou Higher Education Mega Center, Guangzhou 510006, China; c Engineering and Technological Research Centre of Guangdong Province on Intelligent Sensing and Process Control of Cold Chain Foods, & Guangdong Province Engineering Laboratory for Intelligent Cold Chain Logistics Equipment for Agricultural Products, Guangzhou Higher Education Mega Centre, Guangzhou 510006, China; d Food Refrigeration and Computerized Food Technology (FRCFT), Agriculture and Food Science Centre, University College Dublin, National University of Ireland, Belfield, Dublin 4, Ireland

**Keywords:** Box-Behnken design, Reactive species, Foodborne illness, Lipid peroxidation, Cavitation

## Abstract

A novel method of thermoultrasound-assisted plasma functionalized buffer (PFB) for decontaminating grass carp was evaluated using the Box–Behnken design (BBD) with processing variables including PFB generating voltage (P_V_), ultrasound treatment time (U_T_) and temperature (T_P_). The predicted models were found to be significant (*p* < 0.05) and displayed sufficient fitness with experimental data as indicated by non-significant (*p* > 0.05) lack of fit and high coefficient of determination (R^2^≥0.97) values. The optimum decontamination conditions for the responses of *S. putrefaciens* and *S.* Typhimurium were P_V_ of 66 V, U_T_ of 14.90 min and T_P_ of 60 ℃, achieving reductions of 4.40 and 3.97 log CFU/g, respectively, with a desirability of 0.998. Among the variables, temperature presented higher significance for inactivating bacteria and the production of volatile basic nitrogen and lipid peroxidation under the optimized conditions were within the limits of freshness for grass carp. Additionally, the effects of PFB and the optimized thermoultrasound-assisted PFB decontamination were mild on the microstructure of grass carp with slight ruptures and loose myofibril structures, indicating the potential of thermoultrasound-assisted PFB for seafood products decontamination with reduced processing time.

## Highlights

• Thermoultrasound-assisted PFB decontamination was optimized for bacteria reduction.
• Optimized conditions yielded higher reductions compared with individual treatments.
• Predictive models had adequate fitness with temperature variable more significant.
• Lipid oxidation and TVB-N after treatment were within limits of fish freshness.
• Decontamination was mild on fish microstructure and myofibril degradation.

## 1 Introduction

The safety and quality of seafood products have become a challenging preoccupation from both the public health and international trade perspectives [1], [2], [3], as pathogen-contaminated popular freshwater fish like grass carp could serve as an effective medium for foodborne illness outbreaks with significant health threats, due to neutral pH, excellent nutritional properties and high water activity [4]. *Shewanella putrefaciens* (*S. putrefaciens*) and *Salmonella* Typhimurium (*S.* Typhimurium) have been identified as the predominant seafood spoilage microorganisms, and they can be acquired from polluted waters and contaminated handling and processing surfaces [5], [6]. Therefore, effective decontamination approaches are necessary for improving the microbial safety of seafood products and minimizing health hazards.

Conventional industrial water washing with or without chemical sanitisers is a popular practice for reducing the microbial load of fish, but the formation of disinfection by-products, regulatory issues and undesirable changes in product quality have limited their widespread use [7]. Among the developing seafood decontamination methods, the functionalization of liquids and solutions of buffers and organic acids by cold plasma presents promising broad-spectrum washing disinfectants, whose biochemical activities are attributed to a unique blend of highly reactive oxygen and nitrogen species (RONS) [8], [9], [10], [11], [12], [13]. For example, the inherent antimicrobial properties of lactic acid were enhanced for reducing the population of *Salmonella* Enteritidis spot-inoculated on beef when low levels of lactic acid in water was functionalized by cold plasma [14]. In our previous study, the potential of cold plasma technology to functionalize citrate–phosphate buffer to form plasma functionalized buffer (PFB) for decontaminating fish was also demonstrated with encouraging results [12].

However, for further improving food safety, the hurdle technology of combining functionalized liquids and ultrasound have been studied with enhanced inactivation efficiency [15], [16], [17], [18]. In such a hurdle technology, high-intensity ultrasound (20–100 kHz) induces cavitation from the growth and collapse of bubbles, which generates localized rapid pressure and extreme heating, leading to elevated shear actions that facilitate removal of microorganisms from the surface of samples [19], [20], [21]. In addition, pyrolysis reactions that generate RONS have been reported to be triggered by ultrasound-induced decomposition of water [18], while the combination treatment of heat and ultrasound has also been demonstrated to be more effective for microbial inactivation when compared with the use of ultrasound alone [22]. Consequently, the decontamination efficacy of PFB should be enhanced by thermoultrasound as the combination of shear actions from ultrasound and heat can provide additional reduction and expedite the actions of PFB.

Despite the great potential of the hurdle technology in enhancing the effectiveness of microbial decontamination, limited studies exist on the application of PFB as a sanitiser and thermoultrasound-assisted PFB has not been explored for the decontamination for seafood products. In addition, for the hurdle technology to be considered for industrial applications, optimization of the processing conditions is necessary in order to minimize treatment time for obtaining the desired microbial reduction levels while maintaining quality. Response surface methodology (RSM) is a widely applied technique for accessing several factors and their interactions with the aim of optimizing responses, and the Box–Behnken quadratic design (BBD) is frequently utilized as it requires fewer design points [20], [23]. Therefore, in the current study, the effects of thermoultrasound-assisted PFB decontamination on the reduction of *S. putrefaciens* and *S.* Typhimurium population on grass carp were investigated. The Box-Behnken quadratic design (BBD) was employed for optimizing the key process variables including PFB generating voltage (P_V_), ultrasound treatment time (U_T_) and temperature (T_P_), and quality properties and microstructure of the grass carp treated under the optimized conditions were also evaluated.

## 2 Materials and methods

### 2.1 Chemicals and reagents

Sodium chloride (NaCl), n-hexane (C_6_H_14_), glutaraldehyde (C_5_H_8_O_2_) and *para*-anisidine (C_7_H_9_NO) were procured from Shanghai Aladdin Biochemical Technology Co., Ltd. (Shanghai, China). Glycerol (C_3_H_8_O_3_), ethanol (CH_3_CH_2_OH) and glacial acetic acid (CH_3_CO_2_H) were purchased from Tianjin Fuyu Fine Chemical Co., Ltd. (Tianjin, China). Sodium hydrogen phosphate (Na_2_HPO_4_), sodium dihydrogen phosphate (NaH_2_PO_4_), citric acid (C_6_H_8_O_7_), soluble starch (C_6_H_10_O_5_)n and methyl red (C_15_H_15_N_3_O_2_) were supplied by Aladdin Industrial Co. (Shanghai, China). Sodium thiosulphate (Na_2_S_2_O_3_), chloroform (CHCl_3_), perchloric acid (HClO_4_) and hydrochloric acid (HCl) were obtained from Guangzhou Rongman Technology Co., Ltd. (Guangzhou, China). Sodium hydroxide (NaOH), boric acid (H_3_BO_3_) and potassium iodide (KI) were purchased from Sinopharm Chemical Reagent Co., Ltd. (Shanghai, China), and bromocresol green (C_21_H_14_Br_4_O_5_S) was bought from Shanghai Macklin Biochemical Co., Ltd. (Shanghai, China). Nutrient agar (NA), xylose lysine deoxycholate (XLD) agar, Luria-Bertani (LB) broth and agar were provided by Huankai Microbial Science and Technology Co., Ltd. (Guangzhou, China).

### 2.2 Bacteria strains and culture preparation

Bacteria strains of *S*. *putrefaciens* ATCC BAA-1097 and *S.* Typhimurium ATCC14028 supplied by Guangzhou Microbial Culture Centre (Guangzhou, China) were used for the study and stock cultures were stored in LB broth containing 50% C_3_H_8_O_3_ at −80 ℃. To obtain working culture, each strain was cultured twice at 37 ℃ for 18–24 h in the LB broth and streaked onto a plate containing nutrient agar and LB agar, respectively for *S*. *putrefaciens* ATCC BAA-1097 and *S.* Typhimurium ATCC14028. The plates were placed in an incubator (LRH – 70F, Shanghai Qixin Scientific Instrument Co., Ltd, Shanghai, China) at 37 ℃ for 18–24 h, and then examined for confirming the formation of typical and homogenous colony morphology, which was immediately used for inoculum preparation.

### 2.3 Sample preparation and bacteria inoculation

The concentrations of *S*. *putrefaciens* and *S.* Typhimurium used for inoculation were 6–7 log CFU/mL, and the inoculum was prepared in LB broth by incubation at 37 ℃ with agitation on an orbital shaker (WSZ-10A, Shanghai Yiheng Technology Co., Ltd., Shanghai, China) at 150 rpm for 24 h. The cell suspensions of each strain were centrifuged (JW-3024HR, Anhui Jiaven Equipment Industry Co., Ltd., Hefei, China) at 3380 × g and 4 ℃ for 10 min, washed twice and re-suspended in sterile 0.85% NaCl solution. The bacterial cell count of each inoculum was determined by plating 0.1 mL from 10-fold serial dilutions on NA and XLD agar for *S*. *putrefaciens* and *S.* Typhimurium, respectively, and incubated for 24 h at 37 ℃. Fresh grass carp were purchased at a local supermarket (Guangzhou, China) and transported in an iced box to the laboratory after evisceration and filleting. Samples of 10 g obtained from the fillets were separately inoculated with *S*. *putrefaciens* and *S.* Typhimurium by spreading 0.1 mL of each inoculum on the surface. Inoculated samples spread on aluminium foil were dried for 1 h in a laminar flow hood (BSC-1100IIB2-X, Jinan Biobase Biotech Co., Ltd., Jinan, China) at room temperature of 25 ℃ with the fan running to allow for bacteria attachment. The dried inoculated samples with bacteria concentrations of 5–6 log CFU/g were immediately used for decontamination studies.

### 2.4 Generation and characterization of PFB

The dielectric barrier discharge (DBD) atmospheric cold plasma system described in previous studies [12], [24] was used to generate PFB. To prepare PFB, 20 mL of citrate–phosphate buffer prepared by mixing 4.29 g Na_2_HPO_4_ and 11.01 g C_6_H_8_O_7_ in 500 mL of double-distilled water (DDW) was functionalized by DBD cold plasma for 8 min at a gas distance of 5 mm between the solution surface and upper electrode and input voltages of 50, 60 and 70 V, which was designated as PFB50, PFB60 and PFB70, respectively. The PFB (PFB50 - PFB70) was then transferred to sterile falcon tubes for the analysis of physicochemical properties. The temperature of the PFB was measured with an infrared thermal imaging camera (FLIR E5, FLIR Systems AB, Taby, Sweden), the pH and oxidation–reduction potential (ORP) were measured with a multi-parameter meter (PHS-3C, Shanghai Inesa Instrument Co, Ltd., Shanghai, China), while the electrical conductivity (EC) was determined using a conductivity meter (DDS-11A, Shanghai Leici-Chuangyi Instruments & Meter Co. Ltd., Shanghai, China). The ozone (O_3_) level and presence of hydrogen peroxide (H_2_O_2_) were estimated by an ozone meter (DOZ30, Chuangyue Environmental Protection Technology Co., Ltd., Guangzhou, China) and a H_2_O_2_ assay kit (Yuanye Biotechnology Co. Ltd., Shanghai, China), respectively, while the concentration of nitrites and nitrates were determined from the method of Shen et al. [25].

### 2.5 Experimental design and response surface modelling

Preliminary investigation of the suitable ranges of independent variables of PFB generating voltage (P_V_), ultrasound treatment time (U_T_) and temperature (T_P_) was performed using single-factor experiments, and RSM was subsequently used to evaluate the combined performance of the three variables in reducing bacteria on grass carp. The BBD was applied to determine the optimal conditions of thermoultrasound-assisted PFB decontamination of *S*. *putrefaciens* and *S.* Typhimurium on grass carp. The respective ranges of the experimental independent variables of P_V_, U_T_ and T_P_ were selected and coded according to the following equation:

(1) $$K_{i}=\frac{k-\left[k_{a}+k_{b}\right]/2}{\left[k_{a}-k_{b}\right]/2}$$

where $K_{i}$, k, $k_{a}$ and $k_{b}$ represents the coded variable, natural variable, maximum natural variable, and minimum natural variable, respectively. The minimum and maximum levels of P_V_ (K_1_), U_T_ (K_2_), and T_P_ (K_3_) are listed in Table 1.

**Table 1:** Independent variables and their levels for thermoultrasound-assisted PFB decontamination.

Independent variables	Unit	Symbols	Coded levels		
			−1	0	+1
PFB generating voltage, *P_V_*	V	K_1_	50	60	70
Ultrasound treatment time, *U_T_*	min	K_2_	5	10	15
Temperature, *T_P_*	℃	K_3_	50	55	60

The experimental data for the reduction of *S*. *putrefaciens* and *S.* Typhimurium from BBD were analyzed based on multiple regression to fit the following second-order polynomial model:

(2) $$Y=b_{o}+\sum_{i=1}^{n}b_{i}K_{i}+\sum_{i<j}^{n}b_{\mathrm{ij}}K_{i}K_{j}+\sum_{i=1}^{n}b_{\mathrm{ii}}K_{i}^{2}$$

where Y is the response or dependent variable of bacteria log reduction, n is the number of variables, $b_{o}$, $b_{i}$, $b_{\mathrm{ij}}$ and $b_{\mathrm{ii}}$ are constant coefficients of intercept, linear, interaction, and quadratic effects, respectively. $K_{i}$ and $K_{j}$ are levels of independent variables.

### 2.6 Individual and assisted decontamination with PFB, ultrasound, and heat

Individual decontamination by PFB was achieved by immersing inoculated samples in 20 mL of each PFB (PFB50, PFB60 and PFB70) in sterile falcon tubes positioned on the orbital shaker at 150 rpm for 5 min and room temperature of 25 ℃. Ultrasound treatment was performed at room temperature from a bath-type sonochemical reactor (SB25-12D, Ningbo Xinzhi Ultrasonic Equipment Co., Ltd., Ningbo, China) equipped with a rectangular tank with internal dimensions of 500 x 300 x 150 mm (L x W x H), operating at a frequency of 40 kHz and acoustic power of 500 W. Samples were immersed in sterile falcon tubes containing 20 mL DDW fixed on a tube rack, positioned at the centre equidistant from the walls of the tank and treated for 5, 10 and 15 min, designated as US5, US10 and US15, respectively. The tubes were immersed to a depth of 80 mm from the bottom of the tank which corresponded with the height of water in the tank to give a volume of 12 L (500 x 300 x 80 mm: L x W x H) during operation. The temperature of the system after treatment times of 5, 10 and 15 min were 26.10, 27.25 and 28.40 ℃, and were used to determine the effective acoustic intensity of 15.35, 15.70 and 15.82 W/L dissipated by the reactor for each treatment time, respectively, from the calorimetric method expressed below [26]:

(3) $$P_{\mathrm{diss}}=mC_{p}\left(\frac{\mathrm{dT}}{\mathrm{dt}}\right)$$

(4) $$Acoustic\;intensity=\frac{P_{\mathrm{diss}}}{V}$$

where $P_{\mathrm{diss}}$ is ultrasonic power dissipated into the water in the tank, m is mass of the water in the tank (kg), $C_{p}$ is the specific heat capacity of water (4187 J/kg K), (dT/dt) is the slope of the temperature versus time curve for each treatment time of 5, 10 and 15 min, and V is the volume of water in the tank (L).

For heat treatment, samples were immersed in a sterile falcon tube containing 20 mL DDW and placed in a thermostat water bath (Changzhou Aohua Instrument Co., Ltd., Changzhou, China) for 15 min at 50, 55 and 60 ℃, which were designated as T50, T55 and T60. For the thermoultrasound-assisted PFB treatment, samples were first subjected to ultrasound treatment as described above at 50, 55 and 60 ℃ for 5, 10 and 15 min (thermoultrasound) as primary decontamination and subsequently immersed in PFB for 5 min as secondary decontamination.

### 2.7 Conventional water washing (CDW)

Inoculated samples were immersed in 20 mL DDW and placed on the orbital shaker for 5 min at 150 rpm and room temperature of 25 ℃ to emulate an industrial water washing set up, which was designated as CDW. For comparing the quality properties of grass carp at optimized decontamination conditions, CDW was carried out at the optimized treatment time of 14.90 min.

### 2.8 Enumeration of bacteria

Decontaminated samples were added to 90 mL 0.85% sterile NaCl solution in stomacher bags (Huankai Microbial Science and Technology Co., Ltd., Shanghai, China) and homogenized in a stomacher (QIQIAN-08, Qiqian Electronic Technology Co., Ltd., Shanghai, China) for 60 s. A portion of 1 mL of homogenized solutions was used for 10-fold serial dilutions in sterile NaCl and 0.1 mL of serially diluted solutions were spread-plated in triplicates on NA and XLD agar for *S*. *putrefaciens* and *S.* Typhimurium, respectively*.* The media plates were incubated for 24 h at 37 ℃ and bacteria colonies were enumerated and expressed as log CFU/g.

### 2.9 Evaluation of quality properties

The effects of the optimized decontamination conditions on the texture, volatile basic nitrogen and lipid peroxidation of the treated grass carp samples were evaluated and compared with untreated samples and CDW.

#### 2.9.1 Hardness evaluation

Samples were subjected to two consecutive strain cycles at 15% from a 50 mm cylindrical aluminium probe using a texture analyzer (TA.XTplusC, Stable Micro Systems Ltd., Surrey, UK), with triggering force, pre-test, test and post-test speeds of 0.05 N, 2, 5, and 5 mm/s, respectively [27], [28]. The hardness was evaluated from the force–time profile of the Exponent Connect 7.0.6.0 software.

#### 2.9.2 Total volatile basic nitrogen (TVB-N)

The total volatile basic nitrogen (TVB-N) was obtained as per procedure detailed in Cheng et al. [29] with modifications. The filtrate from mincing 5 g of the sample with 45 mL HClO_4_ (0.6 M) was distilled in a Kjeltec distillation unit (8100, FOSS Tecator, Hillerod, Denmark) for 5 min after mixing with 50 mL of NaOH (40%). Two drops of combined indicator of 0.1 g C_15_H_15_N_3_O_2_ and 0.1 g C_21_H_14_Br_4_O_5_S in 100 mL of 93% CH_3_CH_2_OH were added to the distillate in 50 mL of 40 g/L H_3_BO_3_ and titrated with 0.01 M HCl until a faint pink colour appeared, and the TVB-N was expressed as mg N/100 g according to the equation below:

(5) $$TVB-N=\frac{\left(S-B\right)*C*14}{m}*100$$

where S and Bare the volumes (mL) of the titrant for the samples and blank during titration, respectively, C is the concentration of HCl (M) and m is the mass of the sample (g).

#### 2.9.3 Lipid peroxidation

The lipid peroxidation was measured in terms of peroxide value (PV), p-anisidine value (AnV) and total oxidation (totox) from the method described in Okpala [30] with slight modifications. For PV, drops of fresh saturated KI were added to the filtrate from mincing 5 g of sample with 7.5 mL CH_3_CO_2_H and 5 mL CHCl_3_, and kept in the dark at room temperature of 25 °C for 5 min. Iodine was released from the mixture by adding 50 mL DDW followed by titration with 0.01 N Na_2_S_2_O_3_ until the yellow colour disappeared. The titration continued until the blue colour disappeared from the subsequent addition of 0.5 mL starch solution (1%) and the PV was expressed in mEq peroxide/kg of fish according to the equation below:

(6) $$\mathrm{PV}=\frac{\left(S-B\right)*N}{m}*1000$$

where S and Bare the volumes (mL) of the titrant for samples and blank during titration, respectively, N is the normality of Na_2_S_2_O_3_ (mEq/mL), 1000 is the conversion of units (g/kg), and m is the mass of the sample (g).

For AnV determination, the absorbance of the filtrate from homogenizing 5 g of sample with 25 mL C_6_H_14_ and subsequent addition of 1 mL of 0.5% C_7_H_9_NO (2.5 g/L in CH_3_CO_2_H) was measured after 10 min from a UV spectrophotometer (UV-1800, Shimadzu Co., Kyoto, Japan) at 350 nm and the AnV was calculated with the equation below:

(7) $$\mathrm{AnV}=\frac{\left[25*\left\{1.2A_{2}-A_{1}\right\}\right]}{m}$$

where 25 represents volume of C_6_H_14_ used to dissolve the sample, 1.2 represents correction factor for dilution of sample solution with anisidine reagent dissolved in CH_3_CO_2_H, $A_{1}$ and $A_{2}$ are the absorbances before and after adding *para*-anisidine to the filtrate, respectively, and m is the mass of the sample (g),

The totox value was calculated using the PV and AnV values as given below:

(8) Totoxvalue=2PV+AnV

### 2.10 Microstructure observation by scanning electron microscopy (SEM)

For observing the microstructure, slabs of 2.5 mm × 2.5 mm × 2 mm obtained from fresh and decontaminated grass carp were fixed overnight in 2.5% (v/v) C_5_H_8_O_2_ containing 0.01 M phosphate buffer (PBS, pH 7.4) at 4 ℃. The solution was replaced with 0.01 M PBS alone and allowed contact with samples for 30 min. Thereafter, stepwise dehydration of the slabs was carried out in graded concentrations of ethanol (30, 50, 70, 80, and 90%) for 15 min in each solution, followed by dehydration in 100% ethanol twice for 20 min. Dehydrated slabs were vacuum lyophilized (SCIENTZ-18 N, Ningbo Xinzhi Bioscience Co., Inc., Ningbo, China) and coated with 5 nm gold film prior to imaging at 5 kV from high-resolution Merlin SEM (Zeiss Merlin Field Emission SEM, Carl Zeiss NTS GmbH, Oberkochen, Germany).

### 2.11 Statistical analysis

Statistical analysis was performed using Design Expert 11.1.0 (State-Ease Inc., Minneapolis, MN, USA), SigmaPlot 12.0 (Systat Software Inc., CA, USA) and Statistix 9.1 (Analytical Software, Tallahassee, FL, USA). All experiments were conducted in triplicate and data were presented as mean ± standard error of measurement. The interactive relationship between independent variables and the significance of coefficients were assessed from their probability values. The model suitability was evaluated from the F-test, coefficient of variation (CV), lack-of-fit test and coefficient of determination (R^2^) at 5, 0.1 and 0.01% levels of significance.

## 3 Results and discussion

### 3.1 Plasma chemistry and physicochemical properties of PFB

The DBD system utilized air, which is an abundant source of nitrogen and oxygen, as the processing gas and the optical emission spectra of the system have been described in our previous study [12]. Cold plasma generated excited nitrogen and oxygen species in the buffer at the gas–liquid interface and initiated a series of chemical reactions with water molecules to functionalize the buffer with biochemically-active RONS [31], [32], [33], [34], [35]. As shown in Table 2, the temperature of the solution increased significantly with increasing voltage and reached a peak of 49.75 ℃ at 70 V. High voltage dissociated O_2_ in the air first into oxygen atoms because of its lower electron energy when compared with N_2_ [36], and the oxygen atoms combined with O_2_ molecules in water to form O_3_ as described below, which decreased with increasing voltage from 0.68 to 0.38 mg/L.

(9) $$O_{2}+e^{-}\to 2O+e^{-}$$

(10) $$O+O_{2}\to O_{3}$$

**Table 2:** Physicochemical properties of plasma functionalized buffer.

Voltage (V)	pH	Temperature (℃)	ORP (mV)	EC (mS/cm)	O_3_ (mg/L)	H_2_O_2_ (µM)	NO_2_^–^ (mM)	NO_3_^–^ (mM)
0	3.75 ± 0.01a	25.35 ± 0.55c	414.25 ± 5.85c	5.61 ± 0.23c	0.68 ± 0.06a	0.006 ± 0.002b	0.00c	0.00c
50	2.69 ± 0.02b	44.70 ± 0.70b	600.88 ± 4.33b	7.24 ± 0.08b	0.40 ± 0.04b	1.611 ± 0.132a	2.72 ± 0.20b	9.33 ± 0.35b
60	2.67 ± 0.01b	48.15 ± 1.05ab	614.10 ± 4.50ab	8.15 ± 0.10a	0.36 ± 0.03b	2.041 ± 0.204a	3.18 ± 0.08ab	10.94 ± 0.07a
70	2.60 ± 0.01c	49.75 ± 1.00a	630.79 ± 1.05a	8.72 ± 0.01a	0.38 ± 0.03b	2.257 ± 0.098a	3.38 ± 0.06a	11.94 ± 0.04a

ORP: oxidation–reduction potential; EC: electrical conductivity. Values are mean of triplicate measurements ± standard error of measurements; values with different letters in the same column indicate significant differences at *p* < 0.05.

Dissociation of water molecules also occurred when oxygen atoms contacted water molecules to form OH radicals and H_2_O_2_ as shown below [37]:

(11) $$O+H_{2}O\to 2OH$$

(12) $$O+H_{2}O\to H_{2}O_{2}$$

In comparison with O_3_, the H_2_O_2_ generated increased with increasing voltage to a peak of 2.26 µM. OH is a short half-life specie with little contribution to the antimicrobial properties of PFB, while H_2_O_2_ is a long half-life specie with excellent bactericidal properties that may result in the oxidation of membrane lipids, proteins and DNA in bacteria cell [36], [38], [39]. Prolonged exposure led to an accumulation of energy necessary to dissociate N_2_ in the air to form nitrogen oxides (NO_x_), and the dissolution of NO_x_ in water generated H^+^ and other long half-life species of NO_3_^–^, NO_2_^–^ as shown in the equations given below [9], [36]. These reactions significantly increased the acidity of the buffer solution from 3.75 to 2.60.

(13) $$N_{2}+e^{-}\to 2N+e^{-}$$

(14) N+O→NO

(15) $$\mathrm{NO}+O\to NO_{2}$$

(16) $${\mathrm{NO}}_{2}+{\mathrm{NO}}_{2}+H_{2}O\to {\mathrm{NO}}_{2}^{-}+{\mathrm{NO}}_{3}^{-}+2H^{+}$$

(17) $$\mathrm{NO}+{\mathrm{NO}}_{2}+H_{2}O\to 2{\mathrm{NO}}_{2}^{-}+2H^{+}$$

The generated NO_2_^–^ and NO_3_^–^ increased significantly with increasing voltage with values ranging from 2.7 to 3.38 mM and 9.33–11.94 mM, respectively, and the higher values of NO_3_^–^ when compared with NO_2_^–^ may be explained by the disproportionation (p*K*a = 3.3) of NO_2_^–^ to NO_3_^–^ with successive reactions in an acidic environment [7]. In addition, the pH of PFB was<3.3, in which condition NO_2_^–^ have been reportedly converted to HNO_2_ and unstable isomers of HNO_3_ like peroxynitrous acid (ONOOH) and peroxynitrite ion (ONOO^–^), generated from the reaction of NO_2_^–^ and H_2_O_2_ as detailed below [9]:

(18) $${\mathrm{NO}}_{2}^{-}+H^{+}\to HNO_{2}$$

(19) $$\mathrm{HN}O_{2}+H^{+}\to {\mathrm{NO}}^{+}+H_{2}O$$

(20) $$2HNO_{2}\to NO^{\cdot }+NO_{2}^{\cdot }+H_{2}O$$

(21) $${\mathrm{NO}}_{2}^{-}+H_{2}O+H^{+}\to ONOOH+H_{2}O$$

(22) $$\mathrm{HN}O_{2}+H_{2}O_{2}\to ONOO^{-}+H_{2}O$$

Peroxynitrites exhibit strong hydroxylation and oxidation potentials with biomolecules under physiological conditions and may contribute to the antimicrobial properties of PFB [9], [12]. It is evident that the EC of PFB increased significantly from 5.61 to 8.72 mS/cm with increasing voltage application, while the ORP reached 600.88 mV after 50 V and increased to 630.79 mV after 70 V, indicating the increasing production and concentration of reactive ions between plasma and buffer solution at the gas–liquid interface [40]. Together, the results indicated that RONS could be accumulated in PFB and contribute to the antimicrobial potential of PFB, and the overall levels of RONS in PFB had a strong correlation with the generating voltage.

### 3.2 *PFB, ultrasound, and heat treatment-based reductions of S. Putrefaciens* and *S.* Typhimurium

**Fig. 1:**
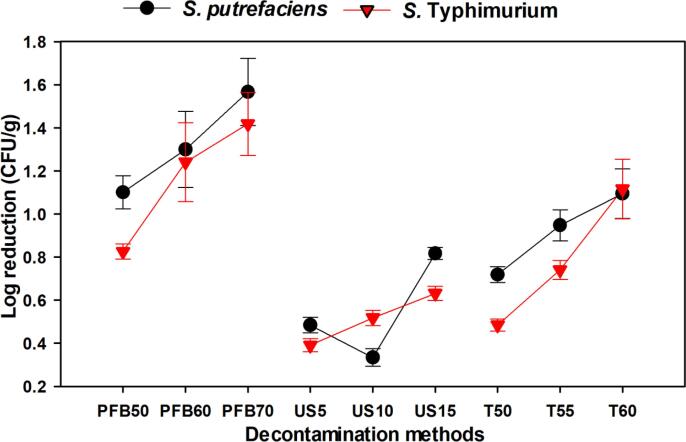
Effects of single treatments of plasma functionalized buffer (PFB), ultrasound and heat on reductions of *S*. *putrefaciens* and *S.* Typhimurium inoculated on grass carp.

Fig. 1 shows the effect of single treatments of PFB, ultrasound, and heat on the reductions of *S. putrefaciens* and *S.* Typhimurium. The initial populations of bacteria were 5.87 and 6.01 log CFU/g, and CDW resulted in reductions of 0.16 and 0.18 log CFU/g for *S. putrefaciens* and *S.* Typhimurium, respectively*.* The reductions of both bacteria increased with increasing generating voltages for samples immersed in PFB, and all voltage levels displayed > 1 log reductions for the target bacteria with the exception of 50 V for *S.* Typhimurium*.* Thus, the highest log reductions were recorded when PFB was generated at 70 V and the values were 1.57 and 1.42 log CFU/g for *S. putrefaciens* and *S.* Typhimurium, respectively. The reduction of target bacteria by ultrasound and heat treatments followed a similar increasing trend with increasing treatment time and temperature, respectively. However single treatments of ultrasound and heat displayed < 1 log reductions for both bacteria, except for temperature application of 60 ℃. The highest reductions for single treatments of ultrasound and heat were 0.82 and 0.63 log CFU/g, and 1.10 and 1.12 log CFU/g for *S. putrefaciens* and *S.* Typhimurium, respectively.

The reductions from thermoultrasound-assisted PFB decontamination are expressed in Table 3 as observed responses, and the data showed increased reductions with increasing levels of the combination of independent variables of PFB generating voltage (P_V_: 50–70 V), ultrasound treatment time (U_T_: 5–10 min) and temperature (T_P_: 50–60 ℃). The reductions ranged from 1.09 to 3.99 log CFU/g and 0.98–3.57 log CFU/g, respectively for *S. putrefaciens* and *S.* Typhimurium, and the highest log reductions were recorded at the maximum levels of the variables to yield 3.99 and 3.57 log CFU/g reductions, respectively for *S. putrefaciens* and *S.* Typhimurium.

**Table 3:** Matrix for independent variables and their corresponding observed and predicted response data from the Box-Behnken quadratic design.

Runs	Independent variables			Responses (Log CFU/g)			
	*P_V_* (V)	*U_T_* (min)	*T_P_* (℃)	*S. putrefaciens*		*S.* Typhimurium	
	K_1_	K_2_	K_3_	Observed	Predicted	Observed	Predicted
1^cp^	60 (0)	10 (0)	55 (0)	2.22	2.23	1.93	1.94
2	70 (+1)	10 (0)	60 (+1)	3.99	4.17	3.57	3.67
3	60 (0)	15 (+1)	50 (−1)	2.17	2.27	1.53	1.64
4	50 (−1)	10 (0)	60 (+1)	1.67	1.76	1.79	2.00
5^cp^	60 (0)	10 (0)	55 (0)	2.23	2.23	1.94	1.94
6	50 (−1)	10 (0)	50 (−1)	1.62	1.44	0.98	0.88
7	70 (1)	10 (0)	50 (−1)	2.48	2.39	2.54	2.33
8	60 (0)	5 (−1)	50 (−1)	1.54	1.70	1.00	1.22
9	50 (−1)	5 (−1)	55 (0)	1.09	1.12	0.98	0.86
10	70 (+1)	15 (+1)	55 (0)	3.67	3.65	2.90	3.01
11	60 (0)	5 (-1)	60 (+1)	2.57	2.47	2.40	2.29
12^cp^	60 (0)	10 (0)	55 (0)	2.23	2.23	1.94	1.94
13^cp^	60 (0)	10 (0)	55 (0)	2.23	2.23	1.94	1.94
14	70 (+1)	5 (−1)	55 (0)	2.71	2.64	2.55	2.55
15	50 (-1)	15 (+1)	55 (0)	1.75	1.81	1.57	1.57
16^cp^	60 (0)	10 (0)	55 (0)	2.24	2.23	1.94	1.94
17	60 (0)	15 (+1)	60 (+1)	3.75	3.60	3.25	3.03

Physiological and structural deformations, DNA and membrane damages were associated with the exposure of bacteria to mildly lethal temperatures, while the internal shear force, actions of RONS from pyrolysis reactions and pressure gradients from the implosion of cavitation bubbles were linked to bacteria inactivation by ultrasound treatment [20], [41], [42]. Also, analysis of the properties of PFB showed strong acidification, enhanced ORP and EC, and the generation of RONS, peroxynitrous acid (ONOOH) and peroxynitrite anion (ONOO^–^) with increasing plasma generating voltages. The structure and functionality of biological macromolecules have been reported to be affected by high levels of acidity while high ORP can alter the redox state of microorganisms and inactivate their defence mechanism to cause membrane damage [43], [44]. It could thus be inferred that the antibacterial effects of PFB on the target bacteria might be attributed to the synergistic actions of ORP, EC, H_2_O_2_, NO_2_^–^, and NO_3_^–^ in an acidic environment. Thus, DNA and membrane damage from heat treatment and acoustic cavitation-induced chemical reactions from hydroxyl radicals and shear force actions via microstreaming provided additional log reductions to further improve the antibacterial effects of PFB.

The results of the current study have demonstrated that thermoultrasound-assisted PFB decontamination of grass carp produced better log reductions of target bacteria when compared with single treatments of PFB, ultrasound or heat. The USA Food and Drug Administration associates 1, 2, 3, 4, and 5 log reductions with 90, 99, 99.9, 99.99, and 99.999% reductions in biological contaminants, respectively [45]. Thus, the log reductions in the current study corresponds to approximately 99.99% reductions in pathogenic bacteria and presents thermoultrasound-assisted PFB decontamination as an effective and appropriate technique for the decontamination of grass carp. The results also indicated that the dynamics of bacterial reduction varied with species. Previous studies have suggested that the retention of lipopolysaccharides and extra firm peptidoglycans film of gram-positive bacteria can add to their structural strength to profer membrane resistance, and thus it can be expected that gram-positive bacteria are less sensitive to decontamination methods when compared with gram-negative bacteria cells [20], [46]. This could be the reason for the less sensitivity of gram-positive *S.* Typhimurium cells to the decontamination methods in the current study as compared with the higher population log reductions observed with gram-negative *S. putrefaciens* cells.

### 3.3 Response surface model fitting

A total of 17 experimental runs were carried out from the BBD and the observed and predicted responses of log reduction values for *S. putrefaciens* and *S.* Typhimurium are shown in Table 3, and Table 4 presents the estimated ANOVA parameters and significances of the predictive models. The model summary statistics showed that independent variables and responses fitted well to a second-order polynomial model for *S. putrefaciens* and *S.* Typhimurium with R^2^ of 0.98 and 0.97, respectively, and the fitted models are given below:

(23) $$S.putrefaciens:Y=44.1312-0.3367K_{1}-0.3751K_{2}-1.2996K_{3}+0.000028K_{1}^{2}+0.0029K_{2}^{2}+0.0083K_{3}^{2}+0.0016K_{1}K_{2}+0.0073K_{1}K_{3}+0.0056K_{2}K_{3}$$

(24) $$S.Typhimurium:Y=18.4982-0.1106K_{1}+0.0050K_{2}-0.7015K_{3}+0.0012K_{1}^{2}-0.0024K_{2}^{2}+0.0066K_{3}^{2}-0.0012K_{1}K_{2}+0.0011K_{1}K_{3}+0.0032K_{2}K_{3}$$

**Table 4:** Estimated ANOVA parameters and significances of predictive models for the reduction of *S. putrefaciens* and *S.* Typhimurium.

Factor	DF	*S. putrefaciens*					*S.* Typhimurium				
		CE	SS	MS	F-value	p-value	CE	SS	MS	F-value	p-value
Model	9	2.23	10.12	1.12	48.79	< 0.0001	1.94	8.83	0.9806	27.13	0.0001

Linear											
$K_{1}$(*P_V_*)	1	0.8387	5.63	5.63	244.27	< 0.0001	0.7800	4.87	4.87	134.65	<0.0001
$K_{2}$(*U_T_*)	1	0.4268	1.46	1.46	63.24	< 0.0001	0.2901	0.6734	0.6734	18.63	0.0035
$K_{3}$(*T_P_*)	1	0.5225	2.18	2.18	94.80	< 0.0001	0.6169	3.04	3.04	84.22	<0.0001

Interaction											
$K_{1}K_{2}$	1	0.0785	0.0246	0.0246	1.07	0.3354	−0.0610	0.0149	0.0149	0.4118	0.5415
$K_{1}K_{3}$	1	0.3650	0.5329	0.5329	23.13	0.0019	0.0555	0.0123	0.0123	0.3409	0.5777
$K_{2}K_{3}$	1	0.1400	0.0784	0.0784	3.40	0.1076	0.0788	0.0248	0.0248	0.6863	0.4348

Quadratic											
${K_{1}}^{2}$	1	0.0028	0.0000	0.0000	0.0015	0.9703	0.1164	0.0571	0.0571	1.58	0.2492
${K_{2}}^{2}$	1	0.0728	0.0223	0.0223	0.9699	0.3575	−0.0588	0.0146	0.0146	0.4031	0.5457
${K_{3}}^{2}$	1	0.2069	0.1802	0.1802	7.82	0.0267	0.1652	0.1149	0.1149	3.18	0.1178

Model adequacy											
Residual	7		0.1613	0.0230				0.2530	0.0361		
Lack of fit	3		0.1612	0.0537	2798.63	0.0816		0.2530	0.0843	9067.86	0.0874
Pure error	4		0.0001	0.00001				0.0000	0.000009		
Corr. total	16		10.28					9.08			
$R^{2}$		0.9843					0.9721				
Adj-$R^{2}$		0.9641					0.9363				
CV (%)		6.42					9.31				

K_1_ = PFB generating voltage, K_2_ = ultrasound treatment time, K_3_ = temperature, DF = degree of freedom, CE = coefficient of regression, SS = sum of squares, MS = mean square, CV = coefficient of variation. respectively signify, degree of freedom.

The models performed significantly at 95% confidence interval (*p* = 0.05) and the high R^2^ suggested an effective correlation between experimental and predicted reduction values, indicating the suitability of the models for describing the relationship between variables [20], [47]. The adjusted coefficient of determination (Adj-R^2^) was used to determine the goodness-of-fit of the models, which was found to be higher than 0.90 for both responses, indicating the exclusion of insignificant terms in the models [48], [49].

The model adequacy summary from ANOVA output indicated that the second-order polynomial model was highly significant with a very low *p*-value (<0.0001 for *S. putrefaciens* and 0.0001 for *S.* Typhimurium). In addition, the quadratic model showed a statistically insignificant lack-of-fit with *p*-values of 0.0816 and 0.0874 at a 95% confidence interval for *S. putrefaciens* and *S.* Typhimurium, respectively. The suitability of a model is typically dependent on the realization of significant regression and non-significant lack-of-fit [20], [49], and the data in the current study suggested adequate representation of the actual relationships between the responses and significant parameters by RSM. Besides, the CV values were also determined to confirm the degree of dispersion of data and the values were <10% for both responses, suggesting precision, reliability and reproducibility of experiments. High CV usually indicates a large disparity among mean values, which may lead to the development of inadequate response models [20], [49]. Therefore, the current analysis showed that both mathematical models were acceptable in describing the results of thermoultrasound-assisted PFB decontamination of *S. putrefaciens* and *S.* Typhimurium on grass carp.

### 3.4 Effects of independent variables on responses

**Fig. 2:**
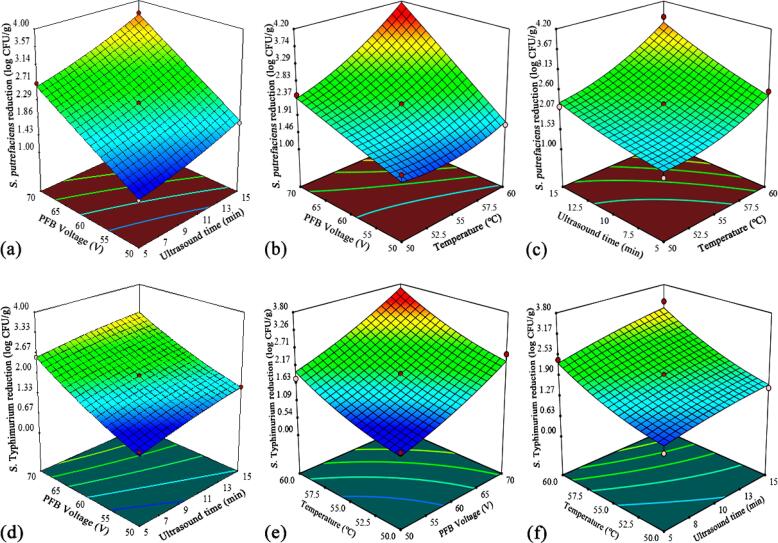
Response surface three-dimensional plots showing the effects of interactions between independent variables on microbial log reductions for *S*. *putrefaciens* (a, b, c) and *S.* Typhimurium (d, e, f).

The effects of independent decontamination variables on responses are also displayed in Table 4. The three variables showed very high significant linear effects (*p* < 0.0001) on the responses with the exception of U_T_ (K_2_) for *S.* Typhimurium response, which displayed a fairly high effect with a *p*-value of 0.0035. Only T_P_ (K_3_) showed a significant quadratic influence at a *p*-value of 0.0267, as displayed for the log reduction of *S. putrefaciens*. Likewise, the interaction effect of variables was only significant for log reduction of *S. putrefaciens* and the significant interaction effect was between P_V_ (K_1_) and U_T_ (K_2_) at a *p*-value of 0.0019. Furthermore, the relationship between independent variables and responses from the predictive Eqs. (12), (13) were visualized as three-dimensional (3D) response contour plots and is presented in Fig. 2. The contour plots illustrated the nature of the interaction between two variables to influence microbial reduction when one variable was fixed at the coded 0 level: (i) P_V_ (60 V); (ii) U_T_ (10 min); and (iii) T_P_ (55 ℃). The interactions for *S. putrefaciens* (Fig. 2a, b, c) were similar to that of *S.* Typhimurium (Fig. 2d, e, f) and the maximum experimental reductions were 3.99 and 3.57 log CFU/g, respectively, at P_V_ of 70 V, U_T_ of 10 min and T_P_ of 60 ℃ as shown in Table 3. Irrespective of P_V_ and U_T_, T_P_ displayed a strong positive interaction with microbial log reductions, which was identified as the most important factor for the reduction of *S. putrefaciens* and *S.* Typhimurium inoculated on grass carp.

### 3.5 Verification of predictive models and validation of optimized conditions

Thirteen treatment sets were selected randomly from the representative 3D response contour plots to test and validate the developed predictive models, and results suggested high correlations of the observed experimental values with the predicted values with R^2^ of 0.97 and 0.95 for *S. putrefaciens* and *S.* Typhimurium, respectively*.* The optimal decontamination conditions for the maximum bacteria reduction were established from the predicted overall desirability index, which points out inconsistencies on the scale of 0–1, indicating absolute undesirable response and maximum desirability [20]. The optimized conditions derived in the current study were P_V_ (K_1_) of 66 V, U_T_ (K_2_) of 14.90 min and T_P_ (K_3_) of 60 ℃, and under these conditions, the maximum reductions of *S. putrefaciens* and *S.* Typhimurium were predicted as 4.40 and 3.97 log CFU/g, respectively, with a desirability of 0.998. Inoculated grass carp were decontaminated under the optimized conditions for validation and reductions of 3.64 and 3.09 log CFU/g were recorded for *S. putrefaciens* and *S.* Typhimurium*,* respectively, which correlated well with the predicted values with an error margin of 1%. Validation results were similar for three replications and the consistent closeness to the predicted values further indicated the reliability and accuracy of the model for replicating the predicted optimization.

### 3.6 Effects of optimized conditions on some quality parameters

The possible effects of thermoultrasound-assisted PFB decontamination under the optimized conditions on the texture, volatile basic nitrogen and lipid peroxidation of grass carp were evaluated and results are presented in Table 5. The hardness of grass carp treated under the optimized conditions was comparable to CDW at 580.76 and 570.67 g, respectively, even though there was an insignificant reduction from the initial value of 594.48 g. The texture of muscle foods is linked with protein contents [50], and the slight reduction in the firmness from initial fresh sample values might be attributed to the degradation of the structural integrity of myofibrils and oxidation-induced water loss from successive actions of heat and RONS from PFB and ultrasound treatment. This was confirmed from the loose myofibril structures and slight ruptures observed during the microstructure analysis of samples decontaminated under optimized conditions, as discussed before.

**Table 5:** Some quality properties of grass carp treated under conventional water washing and optimized thermoultrasound-assisted PFB decontamination conditions.

	Hardness (g)	TVB-N (mg N/100 g)	PV (mEq peroxide/kg)	AnV	Totox
Initial	594.48 ± 4.99a	7.35 ± 0.350b	1.64 ± 0.043b	0.164 ± 0.005b	3.44 ± 0.091b
CDW	580.76 ± 5.11a	7.78 ± 0.095b	1.75 ± 0.056b	0.186 ± 0.009b	3.72 ± 0.121b
TSPFB	570.67 ± 6.17a	14.12 ± 0.445a	3.45 ± 0.312a	0.430 ± 0.035a	7.34 ± 0.589a

CDW: Conventional water washing; TSPFB: Thermoultrasound-assisted plasma functionalized buffer decontamination at optimized conditions; TVB-N: Total volatile basic nitrogen; PV: Peroxide value; AnV: p-anisidine value; Totox: Total oxidation. Values are mean of triplicate measurements ± standard error of measurements. Values with different letters in the same column indicate significant differences at *p* < 0.05.

There was a significant increase in the TVB-N for the samples treated under the optimized conditions, showing a TVB-N value of 12.20 mg N/100 g as compared with 7.78 mg N/100 g for CDW. TVB-N is generally used to assess the freshness of fish and categorized as: <12 mg N/100 g for fresh, 12–20 mg N/100 g for edible but slight deterioration, 20–25 mg N/100 g for borderline and > 25 mg N/100 g for inedible and decomposed [30]. However, Sun et al. [51] considered 15 mg N/100 g as an acceptable and appropriate limit for grass carp, thus the current TBV-N value was within the acceptable limit of freshness for grass carp.

Similar significant increases in PV, AnV and totox were also noticed between samples decontaminated under the optimized conditions and CDW, and the values were 1.75 and 3.45 mEq peroxide/kg, 0.186 and 0.430, and 3.44 and 7.34 for PV, AnV and totox, respectively. The PV typically quantifies the primary oxidation products of peroxides and hydroperoxides, AnV measures secondary lipid oxidation products particularly non-volatile compounds, while totox gives an overall indication of complete oxidation that are the undesirable changes, which may occur due to tissue damages through radical chain mechanism [30], [52], [53], [54]. The PV range for the freshness of fishery products is 5–8 mEq/kg and the AnV for good quality oil is reported to be <2 with a maximum totox of 30 [30], [52], [55]. Therefore, the current results suggested that grass carp treated under the optimized decontamination conditions did not produce adverse effects on lipid peroxidation.

### 3.7 Microstructure analysis

**Fig. 3:**
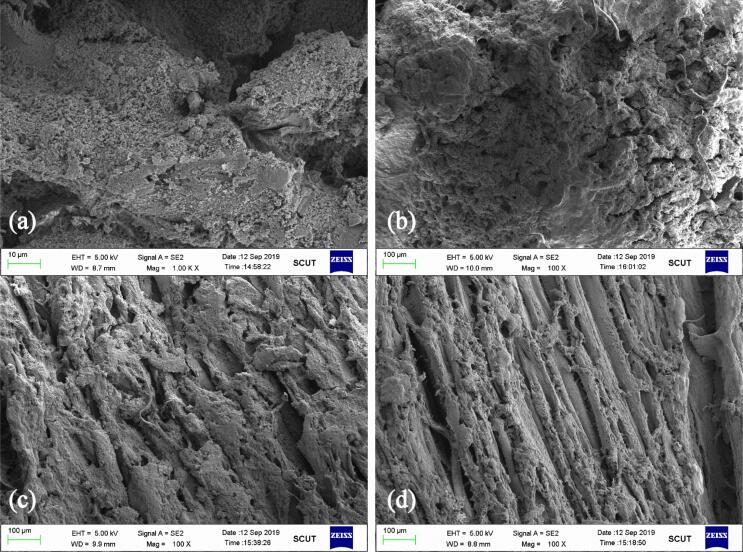
Scanning electron microscopy images of grass carp during exposure to different decontamination methods (a) fresh fillets (b) 5 min immersion in PFB generated at 70 V (c) 15 min ultrasound treatment followed by 5 min immersion in PFB generated at 70 V (d) 15 min ultrasound treatment at 60 ℃ (thermoultrasound) followed by 5 min immersion in PFB generated at 70 V.

The microstructures of fresh and treated grass carp fillets were observed under SEM (Fig. 3), and the microstructure under different treatment conditions revealed differences in intramuscular connective tissues and myofibrils when compared with fresh fillets. The fresh fillets displayed a clear, distinct and dense myofibril structure without any evidence of cracks in the intramuscular connective tissues that were tightly connected to each other. The PFB70 samples exhibited a less dense myofibril structure with the connective tissues still adhering to each other, which was comparable with the untreated samples. Similar observations have been reported for cold plasma treated chub mackerel [56]. However, slight ruptures and loose myofibril structures were observed for samples treated with ultrasound and thermoultrasound prior to immersion in PFB, respectively, indicating slight degradation of microstructure. However, the ruptures were less severe with slight loosely attached connective tissues for samples treated with ultrasound, as compared with samples treated with thermoultrasound prior to immersion in PFB. The presence of ruptures might be attributed to the breakdown of myofibril protein that constitutes intramuscular tissues. Proteases are linked with the hydrolysis of myofibrillar protein, and radial shrinkage of fibres and detachment from surrounding connective tissues are associated with muscle foods at the onset of rigour mortis [56], [57], and thus it was possible that the combined actions of heat, ultrasound and PFB might enhance the activities of proteases and resulting fissures from muscle fibre detachment, causing the ruptures. Besides, the current observations showed that decontamination with PFB produced very mild effects on the myofibrils and microstructure of grass carp compared with thermoultrasound-assisted decontamination.

## 4 Conclusions

The DBD atmospheric cold plasma was demonstrated to be effective for functionalizing and enriching citrate–phosphate buffer solution with abundant RONS, enhancing EC and ORP levels to profer antimicrobial properties. The PFB generated was able to cause significant reductions in the population of *S. putrefaciens* and *S.* Typhimurium when compared with individual treatments of heat and ultrasound. Thermoultrasound and PFB interacted positively with regards to safety and quality maintenance to present improved reductions without adverse effects on the quality and microstructure of grass carp. The BBD was also demonstrated to be an effective and reliable technique for predicting the effects of PFB generating voltage (P_V_), ultrasound treatment time (U_T_) and temperature (T_P_) in reducing target bacteria on grass carp. The quadratic models obtained were satisfactory and accurate for predicting the reductions of bacteria from significant regression coefficients and non-significant lack-of-fit values. Therefore, thermoultrasound-assisted PFB decontamination exhibited promising potential for fish decontamination and could be used as an alternative to conventional water washing. The models reported could serve as an approach to optimize the thermoultrasound-assisted PFB decontamination during seafood processing, while scale-up to pilot decontamination studies may be useful for final validation of the process to meet industrial requirements.

## CRediT authorship contribution statement

**Okon Johnson Esua:** Formal analysis, Investigation, Writing - original draft. **Jun-Hu Cheng:** Validation, Funding acquisition, Resources. **Da-Wen Sun:** Supervision, Funding acquisition, Resources, Writing - review & editing.

## Declaration of Competing Interest

The authors declare that they have no known competing financial interests or personal relationships that could have appeared to influence the work reported in this paper.
